# Spontaneous Reduction in Mitral Valve Regurgitation in Three Dogs With Severe Myxomatous Mitral Valve Disease: An Unusual Case Report

**DOI:** 10.1002/vms3.71155

**Published:** 2026-10-04

**Authors:** Hyo‐Seung Nam, Yein Oh

**Affiliations:** ^1^ Dasom Animal Medical Center Busan Republic of Korea; ^2^ Department of Veterinary Internal Medicine, College of Veterinary Medicine and Institute for Veterinary Biomedical Science Kyungpook National University Daegu Republic of Korea

**Keywords:** coaptation gap, diuretic discontinuation, mitral regurgitation reduction, mitral valve thickening, spontaneous improvement

## Abstract

**Background:**

Myxomatous mitral valve disease (MMVD), the most common heart disease in small‐breed dogs, is characterized by mitral regurgitation (MR) resulting from valvular degeneration, which may progress to left‐sided heart failure. This report describes the cases of three dogs with severe MMVD (American College of Veterinary Internal Medicine Stages C or D) who exhibited an unexpected spontaneous reduction in MR despite continued valve degeneration.

**Case Presentation:**

Three dogs initially presented with symptoms of heart failure and left atrial and ventricular enlargement. Standard treatment, including diuretics, improved their conditions; however, further monitoring revealed that the coaptation gap decreased due to mitral valve thickening, leading to a reduction in MR. This was accompanied by a significant decrease in heart size. As a result, diuretics were discontinued, and all dogs were successfully managed with pimobendan alone.

**Conclusions:**

To our knowledge, this is the first report of spontaneous MR reduction in MMVD, where coaptation gap narrowing played a role in clinical improvement. These cases may broaden the current understanding of MMVD progression, suggesting that, in rare instances, changes in cardiac remodelling and clinical status can occur. Further research is warranted to better understand the underlying mechanisms.

## Graphical abstract

In dogs with MMVD, severe valvular degeneration with chordae tendineae rupture is generally associated with a poor prognosis despite medical management. These three cases demonstrate spontaneous reduction in mitral regurgitation accompanied by decreased coaptation gap, reduced regurgitant volume, reverse cardiac remodelling, discontinuation of diuretics and prolonged survival.

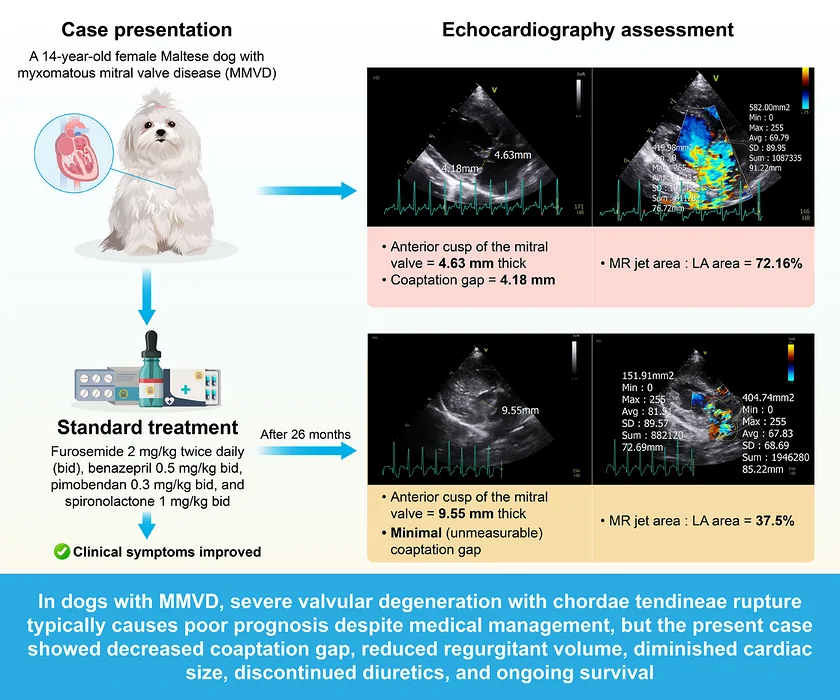

## 1 Introduction

Myxomatous mitral valve disease (MMVD) is the most common heart disease in dogs, especially in small‐breed dogs (Keene et al. 2019). Although the cause of MMVD remains unclear, a genetic component has been implicated in some breeds. This disease is characterized by changes in the cellular components and extracellular matrix of the valve, particularly affecting collagen content and alignment. Expansion of the spongiosa layer is manifested as changes in proteoglycan content. Dysregulation of the extracellular matrix plays a central role, with activated myofibroblasts rapidly breaking down collagen and elastin through increased proteolytic enzyme release (Keene et al. 2019). These mechanisms induce malformation of the mitral apparatus, ultimately leading to mitral regurgitation (MR) (Fox 2012). As a result, cardiac remodelling occurs with left atrial (LA) and left ventricular enlargement, eventually leading to heart failure due to increased LA pressure (Keene et al. 2019). The coaptation gap refers to the ‘gap’ between the anterior and posterior mitral leaflets, representing the area where mitral leaflet coaptation is lacking. The larger the coaptation gap, the greater the regurgitant volume (Adamo et al. 2016).

To alleviate clinical symptoms, the use of diuretics is essential. If mitral valve repair is not performed, the valve condition worsens in most patients, and due to drug resistance and the ceiling effect of diuretics, it eventually progresses to American College of Veterinary Internal Medicine (ACVIM) Stage D, a refractory stage (Keene et al. 2019). In humans, the ceiling dose of furosemide can vary depending on the severity of the patient's condition and the presence of renal failure. Generally, in dogs, the ACVIM Stage D is considered refractory when the total daily dose of furosemide reaches 8 mg/kg or an equivalent dosage of torsemide is being administered (Keene et al. 2019; Oh and Han 2015).

This report documents the cases of three dogs that deviated from the typical progression. All echocardiographic examinations were performed by a single experienced operator using the same ultrasound system (GE Vivid E90). Standard imaging views and measurement protocols were applied consistently across all cases. All cases presented with clinical symptoms related to heart failure and showed partial rupture of the mitral valve chordae on echocardiography, resulting in severe MR. Significant LA and left ventricular enlargement were observed. Standard treatment for left heart failure was administered, and in all cases, clinical symptoms were alleviated. Continuous echocardiographic monitoring revealed further degeneration of the mitral valve, with more pronounced valve thickening. However, as the coaptation gap decreased, a significant reduction in MR was observed. For the evaluation of MR, regurgitant volume (mL) and regurgitant fraction (%) assessment were used as quantitative methods (Figure 1), while the ratio of the MR jet area to the LA area (%) was used as a semi‐quantitative method (Muzzi et al. 2003). As a result, in all three cases, diuretics were discontinued, and the patients are being managed with pimobendan alone. To our knowledge, our report is the first to document spontaneous reductions in MR in cases with ACVIM Stage C or D MMVD with heart failure.

**FIGURE 1:**
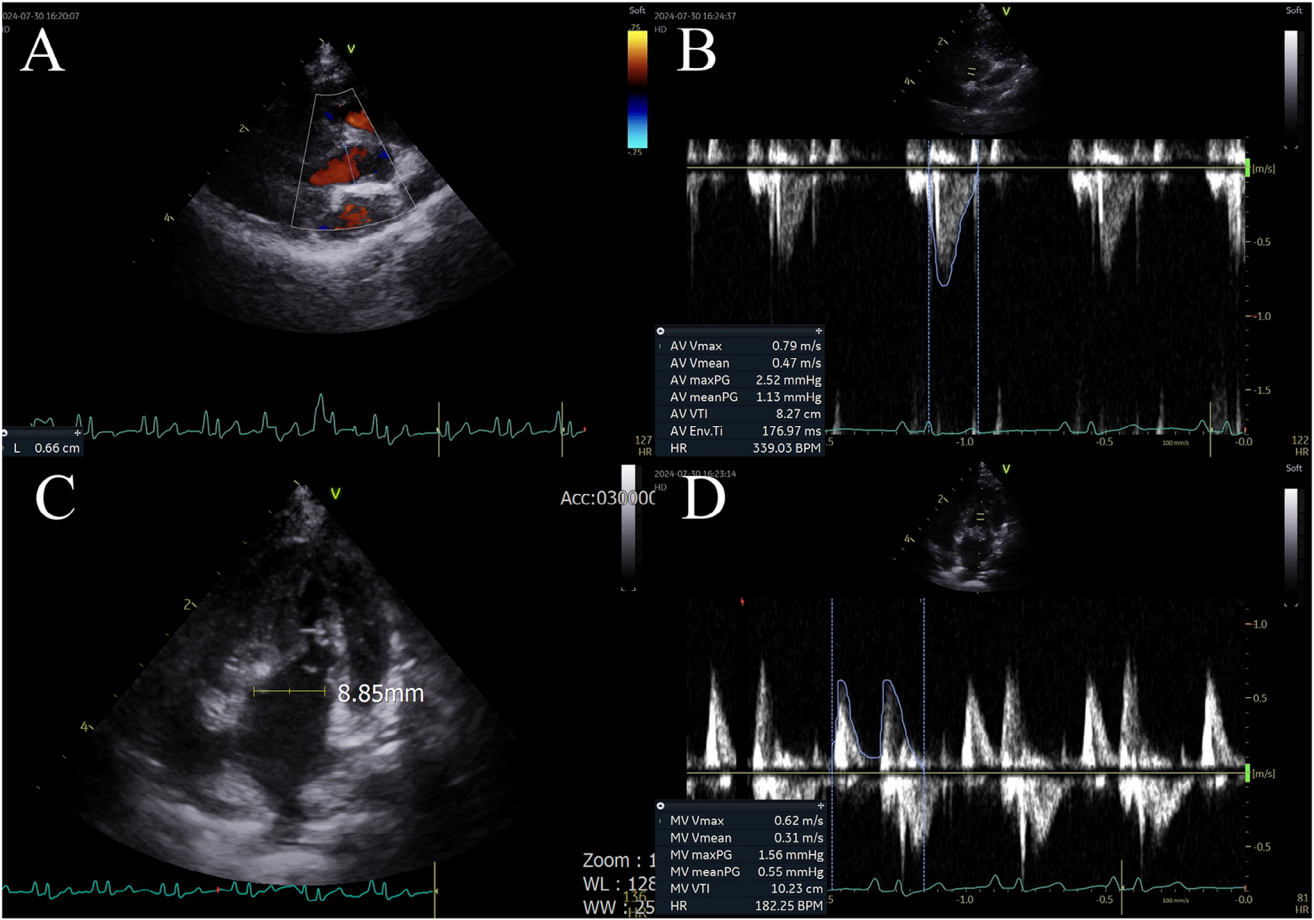
Echocardiographic image taken 28 months after the initial presentation of Case 2. To quantitatively assess mitral regurgitation, both the regurgitant volume and regurgitant fraction were measured. Four key parameters are required for this calculation. (A) The diameter of the aortic valve during systole is measured in the right parasternal five‐chamber view to calculate the forward stroke volume through the aorta. (B) The velocity‐time integral (VTI) of aortic outflow is obtained from the left apical five‐chamber view. (C) To calculate the mitral forward stroke volume during early diastole, the diameter at the tips of the mitral valve leaflets is measured in the left apical four‐chamber view. (D) In addition, the VTI of the E and A waves is measured from the same view (left apical four‐chamber view).

## 2 Case Presentation

### 2.1 Case 1

A 14‐year‐old spayed female Maltese dog presented to our hospital with chief complaints of increased sleeping respiratory rate and coughing. The respiratory rate during sleep had previously remained at 20 breaths per minute, but for more than 3 consecutive days, it was reported to exceed 35 breaths per minute. On thoracic radiographs, the vertebral heart size (VHS) was 12.8, and a vertebral left atrial size (VLAS) of 3.2 indicated LA enlargement. As no distinct pulmonary infiltrates were identified, transthoracic echocardiography was promptly performed. On echocardiography, the thickening of the anterior cusp of the mitral valve was 4.63 mm, the coaptation gap was 4.18 mm and the ratio of the MR jet area to the LA area, representing the semi‐quantitative MR, was 72.16%. The regurgitant volume, which quantitatively measures the amount of MR, was 98.8 mL, and the regurgitant fraction was 95.4%. The LA‐to‐aortic (LA:AO) ratio was 2.52, the left ventricular internal dimension in diastole normalized to bodyweight (LVIDDN) was 2.01, and the E peak was 1.56 m/s, indicating enlargement of both the LA and left ventricle. The patient was started on oral furosemide treatment without prior intravenous administration. After initiating the standard treatment comprising furosemide 2 mg/kg twice daily (bid), benazepril 0.5 mg/kg bid, pimobendan 0.3 mg/kg bid and spironolactone 1 mg/kg bid, the clinical symptoms improved. Subsequently, the diuretic dosage was titrated upward in response to the recurrence of clinical signs, and ultimately, clinical improvement was observed when furosemide was administered at 8 mg/kg/day and torsemide at 0.4 mg/kg/day. Consistent monitoring followed, and 26 months after the initial visit, the patient's heart size was observed to have decreased. Radiographs obtained 26 months after the initial diagnosis showed that the patient's VHS decreased to 11.6 and VLAS decreased to 2.4. On echocardiography, the thickening of the anterior cusp of the mitral valve had increased to 9.55 mm, but the previously observed coaptation gap had decreased to the point that it was difficult to measure. As a result, the ratio of the MR jet area to the LA area (%) had decreased to 37.5%. The regurgitant volume was reduced to 5.67 mL and the regurgitant fraction to 34.5%, compared with previous measurements. The LA:AO ratio was 1.56, LVIDDN was 1.16 and the E peak was 0.85 m/s, showing a reduction compared with previous measurements. The patient has been managed with pimobendan alone for the last 6 months without diuretics (Figure 2).

**FIGURE 2:**
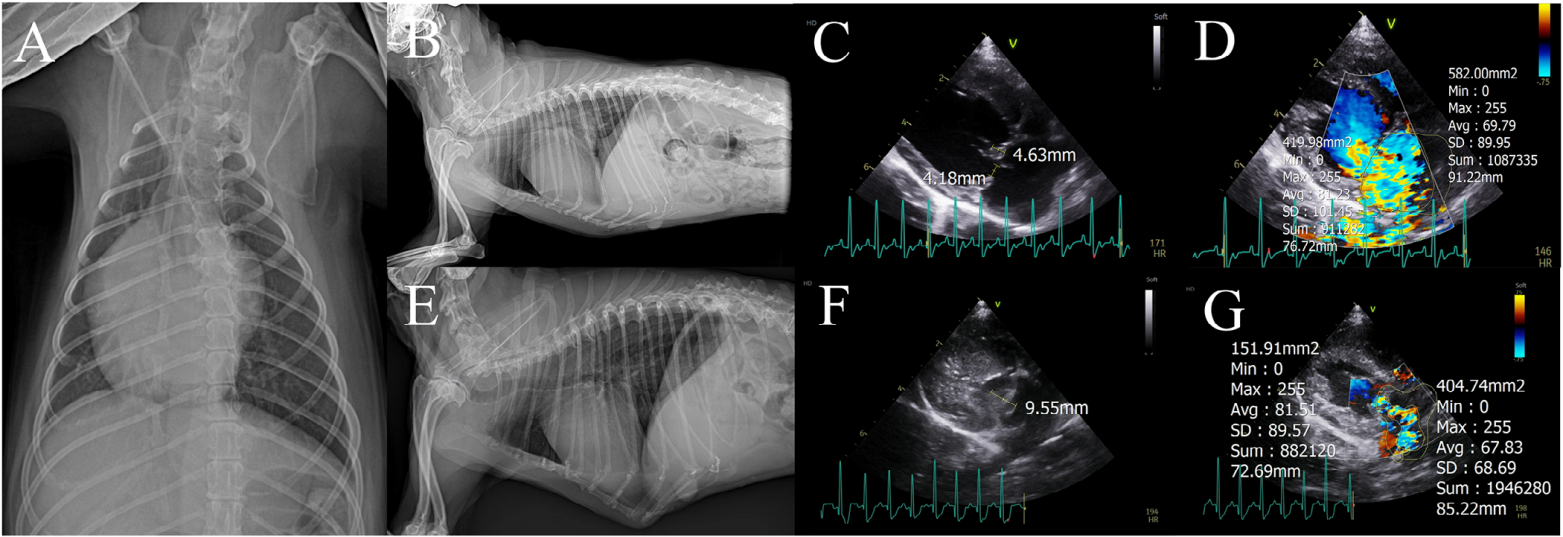
Chest radiographs and echocardiographic images for Case 1. The images labelled A, B, C and D are figures from the initial visit. The images labelled E, F and G are figures from 26 months after the initial visit. (A) In the ventrodorsal chest radiographs, the patient presented with increased respiratory rate during sleep and coughing as the main symptoms, but no remarkable pulmonary infiltrates were observed. (B) In the right lateral chest radiographs, the VHS is 12.8, and LA enlargement is observed. (C) In the right parasternal long‐axis view, partial rupture of the mitral valve chordae is observed and the coaptation gap measures 4.18 mm. The thickness of the anterior cusp of the mitral valve is 4.63 mm. (D) In the right parasternal long‐axis view, the ratio of the MR jet area to the LA area is 72.16%. (E) In the right lateral chest radiographs, the VHS is 11.6, and the size of the LA has decreased compared to that at the initial visit. (F) In the right parasternal long‐axis view, the valve has thickened further to 9.55 mm, but the previously distinct coaptation gap is no longer observed. (G) In the right parasternal long‐axis view, the ratio of the MR jet area to the LA area has decreased to 37.5% compared to the previous measurement. LA, left atrium; MR, mitral regurgitation; VHS, vertebral heart size.

### 2.2 Case 2

A 9‐year‐old castrated male Maltese dog presented to our hospital with dyspnoea and coughing. On thoracic radiographs, the VHS was 11.3, with the VLAS measured at 3.1, which was consistent with LA enlargement. A mild infiltrative lesion was observed in the right caudal lung lobe. After intravenous administration of furosemide at 0.5 mg/kg/h for 8 h and oxygen supplementation, the respiratory rate stabilized. During the subsequent hospitalization period, furosemide was administered intravenously at a dose of 2 mg/kg twice daily, along with continuous oxygen supplementation. On the third day of hospitalization, as the respiratory rate remained stable, a transthoracic echocardiographic examination was performed. On echocardiography, the thickening of the anterior cusp of the mitral valve was 2.78 mm, the coaptation gap was 3.61 mm and the ratio of the MR jet area to the LA area, representing the semi‐quantitative MR, was 88.5%. The regurgitant volume, which quantitatively measures the amount of MR, was 28.33 mL, and the regurgitant fraction was 96%. The LA:AO ratio was 2.13, LVIDDN was 1.55 and the E peak was 1.43 m/s, indicating enlargement of both the LA and left ventricle. Since the echocardiographic examination was performed approximately 2 days after hospitalization and treatment, the echocardiographic parameters may have been underestimated. Similar to the treatment protocol in the first case, the standard treatment comprising furosemide 2 mg/kg bid, enalapril 0.5 mg/kg bid, pimobendan 0.3 mg/kg bid and spironolactone 1 mg/kg bid was initiated, and the patient's clinical condition remained stable. Radiographs obtained 28 months after the initial diagnosis showed that the patient's VHS decreased to 10.0 and VLAS decreased to 2.2. On echocardiography, the thickening of the anterior cusp of the mitral valve had increased to 5.36 mm, but the previously observed coaptation gap had decreased to the point that it was difficult to measure. As a result, the ratio of the MR jet area to the LA area (%) had decreased to 11.1%. The regurgitant volume was reduced to 3.46 mL and the regurgitant fraction to 55.04%, compared with previous measurements. The LA:AO ratio was 1.39, LVIDDN was 1.25 and the E peak was 0.7 m/s, showing a reduction compared with previous measurements. This patient has been managed with only pimobendan for the last 22 months, without any diuretics (Figure 3).

**FIGURE 3:**
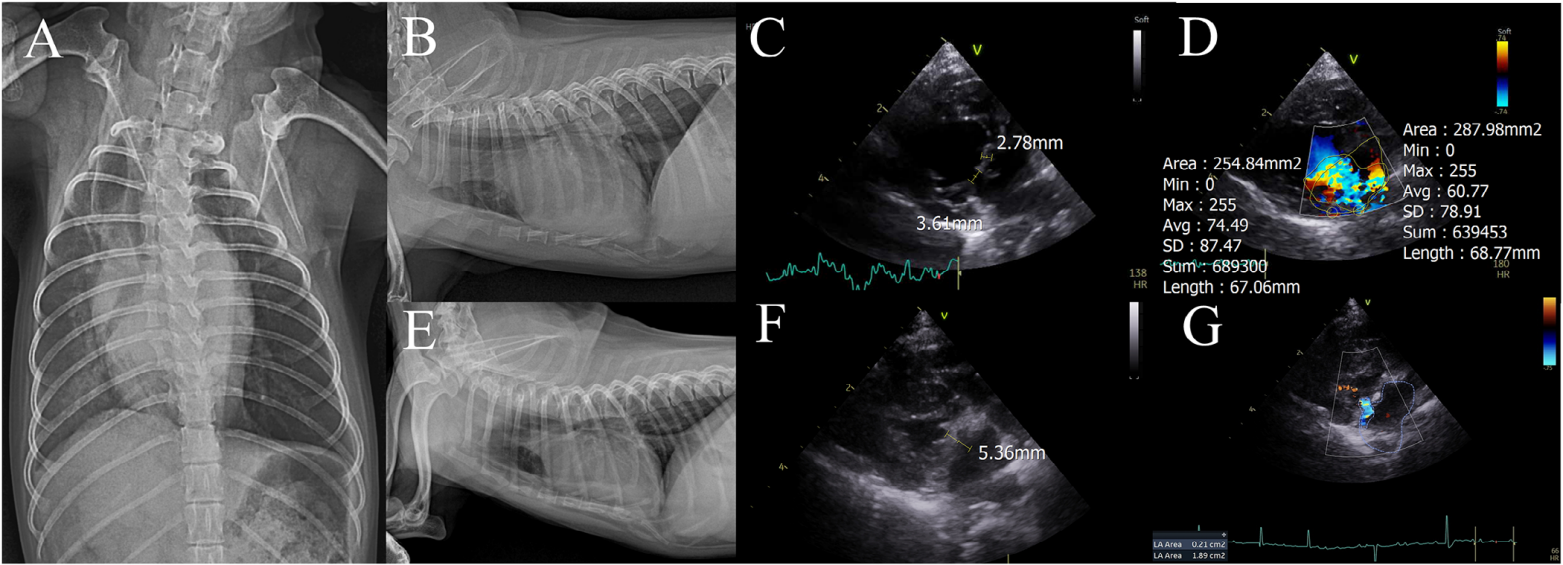
Chest radiographs and echocardiographic images for Case 2. The images labelled A, B, C and D are figures from the initial visit. The images labelled E, F and G are figures from 28 months after the initial visit. (A) In the ventrodorsal chest radiographs, an alveolar pattern observed in the right caudal lung lobe was indicative of mild pulmonary infiltration. (B) In the right lateral chest radiographs, the VHS is 11.3, and LA enlargement is observed. (C) In the right parasternal long‐axis view, the presence of a flail mitral leaflet is indicative of a possible rupture of the chordae tendineae, and the coaptation gap measures 3.61 mm. The thickness of the anterior cusp of the mitral valve is 2.78 mm. (D) In the right parasternal long‐axis view, the ratio of the MR jet area to the LA area is 88.5%. (E) In the right lateral chest radiographs, the VHS is 10, and the size of the LA has decreased compared to that at the initial visit. (F) In the right parasternal long‐axis view, the valve has thickened further to 5.36 mm, but the previously distinct coaptation gap is no longer observed. (G) In the right parasternal long‐axis view, the ratio of the MR jet area to the LA area has decreased to 11.1% compared to the previous measurement. LA, left atrium; MR, mitral regurgitation; VHS, vertebral heart size.

### 2.3 Case 3

A 12‐year‐old castrated male Maltese dog presented to our hospital with dyspnoea and coughing. On thoracic radiographs, the VHS was 11.4, with a VLAS of 3, indicating LA enlargement. A mild infiltrative lesion was observed in the right caudal lung lobe. Clinical signs improved following two intravenous administrations of furosemide at a dose of 2 mg/kg given 2 h apart, along with supplemental oxygen therapy. After clinical improvement, a transthoracic echocardiographic examination was performed. On echocardiography, the thickening of the anterior cusp of the mitral valve was 2.63 mm, the coaptation gap was 2.49 mm and the ratio of the MR jet area to the LA area, representing the semi‐quantitative MR, was 69%. The regurgitant volume, which quantitatively measures the amount of MR, was 82.8 mL, and the regurgitant fraction was 96.36%. The LA:AO ratio was 2.53, LVIDDN was 1.96 and the E peak was 1.91 m/s, indicating enlargement of both the LA and left ventricle. The standard treatment comprising furosemide 1.5 mg/kg bid, enalapril 0.5 mg/kg bid, pimobendan 0.3 mg/kg bid and spironolactone 1 mg/kg bid was initiated, and the patient's clinical condition remained stable. Radiographs obtained 12 months after the initial diagnosis showed that the patient's VHS decreased to 10.8, and the VLAS decreased to 2. On echocardiography, the thickening of the anterior cusp of the mitral valve had increased to 5.37 mm, but the previously observed coaptation gap had decreased to the point that it was difficult to measure. As a result, the ratio of the MR jet area to the LA area (%) had decreased to 27.1%. The regurgitant volume was reduced to 8.91 mL and the regurgitant fraction to 62.61%, compared to previous measurements. The LA:AO ratio was 1.55, LVIDDN was 1.08 and the E peak was 0.63 m/s, showing a reduction compared with previous measurements. This patient has also been managed with only pimobendan since the last 9 months, without any diuretics (Figure 4). The overall heart measurements for each case are listed in Table 1.

**FIGURE 4:**
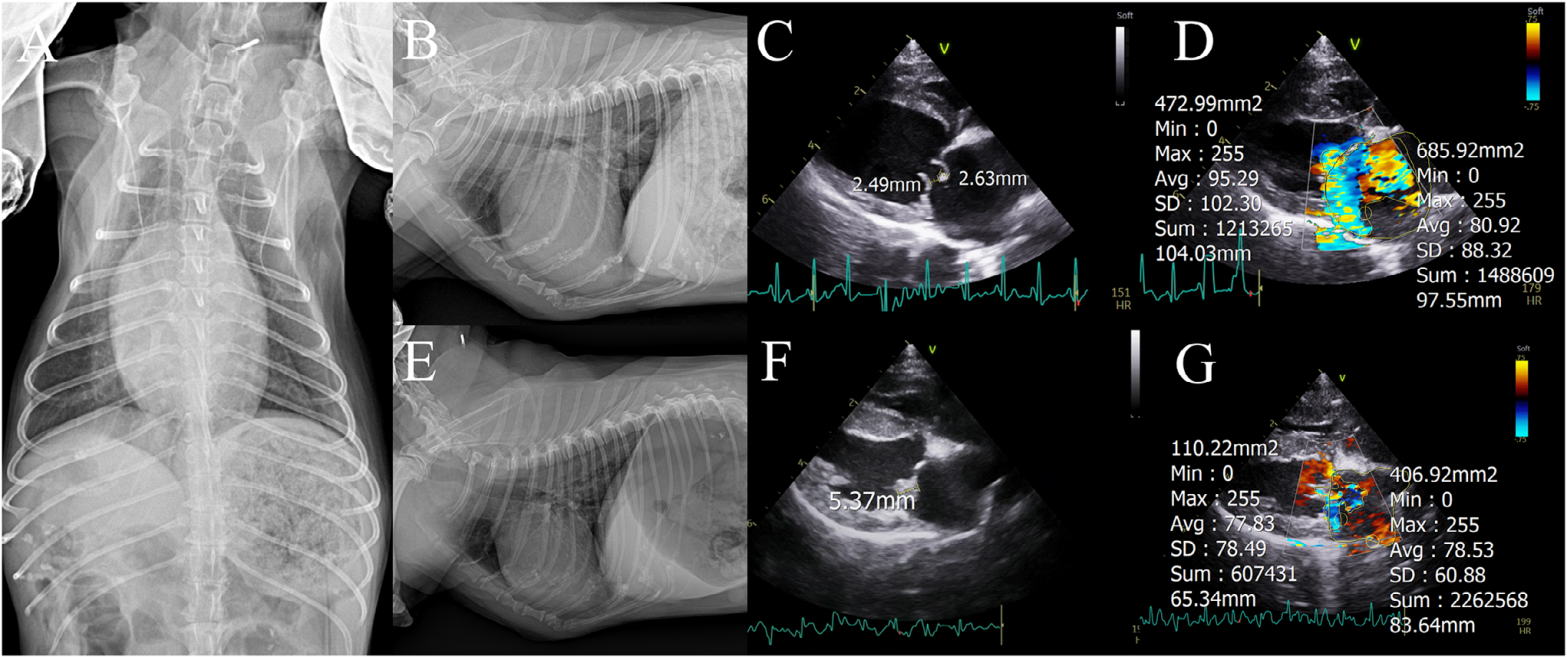
Chest radiographs and echocardiographic images for Case 3. The images labelled A, B, C and D are figures from the initial visit. The images labelled E, F and G are figures from 12 months after the initial visit. (A) In the ventrodorsal chest radiographs, an alveolar pattern observed in the right caudal lung lobe was indicative of mild pulmonary infiltration. (B) In the right lateral chest radiographs, the VHS is 11.4, and LA enlargement is observed. (C) In the right parasternal long‐axis view, the presence of a flail mitral leaflet is indicative of a possible rupture of the chordae tendineae, and the coaptation gap measures 2.49 mm. The thickness of the anterior cusp of the mitral valve is 2.63 mm. (D) In the right parasternal long‐axis view, the ratio of the MR jet area to the LA area is 69%. (E) In the right lateral chest radiographs, the VHS is 10.8, and the size of the LA has decreased compared to that at the initial visit. (F) In the right parasternal long‐axis view, the valve has thickened further to 5.37 mm, but the previously distinct coaptation gap is no longer observed. (G) In the right parasternal long‐axis view, the ratio of the MR jet area to the LA area has decreased to 27.1% compared to the previous measurement. LA, left atrium; MR, mitral regurgitation; VHS, vertebral heart size.

**TABLE 1:** Changes in heart indexes for Cases 1, 2 and 3.

		Case 1	Case 2	Case 3
LA:AO (< 1.70 = Score 1, 1.70–1.90 = Score 2, 1.91–2.5 = Score 3, > 2.50 = Score 4)	Before	2.52	2.13	2.53
	After	1.56	1.39	1.55
LVIDDN (< 1.70 = Score 1, 1.70–2.00 = Score 2, 2.10–2.30 = Score 3, > 2.30 = Score 4)	Before	2.01	1.55	1.96
	After	1.16	1.25	1.08
E peak (m/s) (< 1.20 = Score 1, 1.20–1.50 = Score 2, > 1.5 = Score 3)	Before	1.56	1.43	1.91
	After	0.85	0.7	0.63
Valve thickness (width; mm) (Normal width in 10 kg—1.44 [1.16–1.72])	Before	4.63	2.78	2.63
	After	9.55	5.36	5.37
Coaptation gap (mm)	Before	4.18	3.61	2.49
	After	0	0	0
Regurgitant volume (mL) (mild = 5.39 ± 3.24, moderate = 15.21 ± 10.05, severe = 32.92 ± 20.28)	Before	98.8	28.33	82.8
	After	5.67	3.46	8.91
Regurgitant fraction (%) (mild = 33 ± 11.74, moderate = 57 ± 14.32, severe = 75 ± 8.08)	Before	95.4	96	96.36
	After	34.5	55.04	62.61
Ratio of MR jet area to LA area (%) (< 30% = mild MR, 30%–70% = moderate MR, > 70% = severe MR)	Before	72.16	88.5	69
	After	37.5	11.1	27.1
VHS (No heart enlargement < 10.5)	Before	12.8	11.3	11.4
After	11.6	10	10.8
VLAS (Left atrial enlargement > 3)	Before	3.2	3.1	3
	After	2.4	2.2	2

Abbreviations: LA, left atrium; LA:AO ratio, left atrial‐to‐aortic ratio; LVIDDN, left ventricular internal dimension in diastole normalized to bodyweight; MR, mitral regurgitation; VHS, vertebral heart size; VLAS, vertebral left atrial size.

## 3 Discussion

These cases demonstrate that unexpected clinical outcomes can occur in dogs with severe MMVD. In particular, dogs in ACVIM Stages C and D typically require ongoing medical therapy, including diuretics and other standard treatments, due to progressive disease. However, in the present cases, MR unexpectedly decreased despite this advanced stage. This reduction in regurgitation occurred even as valve degeneration continued, leading to a decrease in the size of the LA and left ventricle, which prompted the discontinuation of diuretic therapy. Although such cases are rare, they suggest that spontaneous improvement in MR is possible, offering hope in the management of severe MMVD. Though rare in human medical literature as well, similar case reports of severe MR caused by a flail mitral valve improving spontaneously have been documented. A flail mitral valve often results in severe regurgitation and potential left ventricular dysfunction if untreated. This case highlights mitral valve adaptation, where anterior leaflet enlargement reduced regurgitation and promoted reverse ventricular remodelling without surgery. This process has been suggested to involve reactivation of embryonic developmental pathways, including endothelial–mesenchymal transformation, leading to increased leaflet area and thickness. Such changes may improve leaflet coaptation and reduce regurgitation (Asmer et al. 2010). However, the exact mechanisms underlying these structural changes in the present cases remain unclear, and the proposed explanations should be interpreted with caution.

MR can occur when the complex interactions of the mitral valve structure are disrupted. In humans, surgical mitral valve repair, introduced by Alain Carpentier in the early 1970s, can reduce MR, leading to dramatic changes in the prognosis and management of patients with severe MR (Lancellotti et al. 2010). The importance of coaptation length after mitral valve repair has been documented (Wei et al. 2017). This importance has also been increasingly emphasized in recent veterinary studies (Mihara et al. 2024). According to the documented evidence, an increase in the coaptation gap can lead to a higher regurgitation volume, and without surgical intervention, the limitations of medical treatment will inevitably become apparent. In addition, through 3D echocardiography, the anatomic regurgitant orifice area was evaluated, which was larger in patients with severe MR than in patients with mild MR (Müller et al. 2017). Although further research is needed on the impact of this variable on diagnosis and prognosis, it can be inferred that the size of the regurgitant orifice itself may influence this pathological condition. These findings are consistent with the present cases, in which reduction of the coaptation gap was associated with decreased MR severity. Notably, in the present cases, reduction of the coaptation gap occurred without any surgical intervention. This observation may have important clinical implications, as it suggests that functional improvement in mitral valve coaptation can, in rare cases, arise through non‐surgical mechanisms in dogs with advanced MMVD.

In addition, in dogs with severe MR, the relatively recent Mitral INsufficiency Echocardiographic (MINE) score has made it easier to predict the severity of MMVD and the median survival duration (Vezzosi et al. 2021). All three cases described in this report showed severe valve degeneration and partial chordae rupture, forming a distinct coaptation gap, with significant regurgitation. In addition, using the MINE score, it was confirmed that these cases were in the severe stage; the expected median survival duration was approximately 623 days (95% confidence interval, 432–710 days). Without mitral valve repair, all of these cases were assumed to have the typically predicted survival duration. However, in the three described cases, despite further progression of valve degeneration, the coaptation gap decreased, as if the patients had undergone mitral valve repair. This was accompanied by reduced left‐sided cardiac dimensions. Currently, the dogs are being managed solely with pimobendan, without any diuretics, and are alive without any notable clinical symptoms of dyspnoea, coughing or syncope.

This exceptional outcome offers new insights into the current understanding of the natural progression of MMVD. The first is the possibility of spontaneous healing. It is noteworthy that in all three cases, despite ongoing degenerative changes in the mitral valve, there was a reduction in MR along with a decrease in the size of the LA and left ventricle. This suggests that the patients achieved clinical stability with medical treatment alone. Disease progression may have halted or even reversed. This phenomenon is difficult to explain with the current knowledge and highlights the need to explore potential mechanisms by which natural reduction in valve regurgitation could alleviate disease progression. Second, the possibility of reversing cardiac remodelling. Recent evidence supports the reversibility of cardiac remodelling in dogs with advanced MMVD (Mihara et al. 2024) demonstrated that surgical mitral valve repair significantly restored coaptation length, reduced regurgitant volume, improved haemodynamics and decreased medication requirements, providing a mechanistic basis that remodelling can be modified even in late‐stage disease. However, the present cases are particularly noteworthy in that they demonstrated spontaneous reverse remodelling without surgical intervention, underscoring the possibility of natural structural recovery in advanced MMVD. One important limitation of this study is the potential confounding effect of medical treatment. Although diuretics were discontinued and the observed improvements were sustained, the contribution of pimobendan cannot be fully excluded. While pimobendan is known to improve haemodynamics, there is currently limited clinical evidence demonstrating its ability to induce marked and sustained reductions in MR severity and cardiac remodelling in advanced MMVD. Therefore, the relative contribution of treatment versus intrinsic structural changes remains uncertain. Third, the connection with long‐term survival. It is also significant that long‐term survival was observed in all three cases after improvement in cardiac remodelling. Typically, in patients with left heart failure due to MMVD, only short‐term survival is expected. However, these three patients survived for several months to years even after discontinuing diuretics. This may be linked to the improvement in cardiac function and provides an important clue about the need to explore new indicators to more accurately predict the prognosis of patients with MMVD. Further research is needed to understand the underlying mechanisms of this phenomenon and identify factors that can help predict such outcomes.

To our knowledge, this is the first report of spontaneous reduction in MR with severe MMVD. It is important to recognize that the prognosis remains poor in MMVD accompanied by chordae tendineae rupture. However, in rare cases, favourable outcomes may occur.

## Author Contributions

**Hyo‐Seung Nam**: conceptualization, investigation, writing – original draft, methodology, visualization, data curation. **Yein Oh**: conceptualization, investigation, writing – review and editing, methodology, supervision.

## Funding

The authors have nothing to report.

## Ethics Statement

The authors confirm that the ethical policies of the journal, as noted on the journal's author guidelines page, have been adhered to. No ethical approval was required as this is a case report.

## Consent

The authors have nothing to report.

## Conflicts of Interest

The authors declare no conflicts of interest.

## Data Availability

The original contributions presented in the study are included in the article, and further inquiries can be directed to the corresponding author.
